# The role of intermittent fasting in the treatment of cognitive dysfunction in type 2 diabetes mellitus

**DOI:** 10.3389/fnut.2025.1603165

**Published:** 2025-05-09

**Authors:** Chunying Cui, Daqing Song, Yan Yang, Xinyu Wang, Renjun Lv, Shanjing Nie, Wenwen Xu

**Affiliations:** ^1^Emergency Department, Jining No.1 People's Hospital, Jining, Shandong, China; ^2^Institute of Emergency and Critical Care of Jining Medical Research Academy, Jining, Shandong, China; ^3^Department of Geriatric Neurology, Shandong Provincial Hospital Affiliated to Shandong First Medical University, Jinan, Shandong, China; ^4^Department of Ophthalmology, Shandong Provincial Hospital Affiliated to Shandong First Medical University, Jinan, Shandong, China

**Keywords:** intermittent fasting, type 2 diabetes mellitus, diabetes-related cognitive dysfunction, insulin resistance, neuroinflammation, gut-brain axis dysregulation, oxidative stress

## 1 Introduction

A pooled analysis of 1,108 population-representative studies published in 2024 noted that type 2 diabetes mellitus (T2DM) affects 828 million adults worldwide (1). Researchers recognize diabetes-associated cognitive dysfunction (DACD) as a critical comorbidity of T2DM, reflecting the intersection of metabolic dysfunction and neurodegeneration. Demographic trends for DACD very closely resemble those seen in diabetes mellitus (2). A systematic review and meta-analysis encompassing >25 original studies with millions of participants, estimates that the relative risk (RR) for all types of cognitive dysfunction is 1.73 (95% CI 1.65–1.82) for people with diabetes compared with people without diabetes (3). DACD shares pathological features overlapping with Alzheimer's disease (AD), including insulin resistance, chronic neuroinflammation, and amyloid-β accumulation (4). While existing therapies focus primarily on glycemic control, few interventions target the brain-specific consequences of T2DM, such as cognitive impairment. The Memory in Diabetes (MIND) sub-study of the Action to Control Cardiovascular Risk in Diabetes (ACCORD) trial is the largest intervention study of cognitive impairment in diabetes mellitus to date, found no benefit of intensive glycemic control on cognitive function (5). Therefore, “novel” interventions to address DACD are urgently needed.

Intermittent fasting (IF), a dietary regimen alternating periods of fasting and feeding, has emerged as a promising intervention to mitigate both metabolic and cognitive deficits in T2DM (6). IF—encompassing regimens like time-restricted feeding and 5:2 fasting (7)—induces metabolic switching from glucose to ketone metabolism, activating pathways that may counteract neurodegeneration (8, 9). The objective of this opinion article is to examine whether IF can improve T2DM-associated cognitive dysfunction by enhancing insulin sensitivity, reducing neuroinflammation, mitigating oxidative stress, and restoring gut microbiota homeostasis, and thoroughly analyzed the potential challenges associated with the clinical translation of IF.

## 2 Pathological links between T2DM and cognitive dysfunction

Evidence exists of a link between type 2 diabetes mellitus (T2DM), cognitive decline, and dementia (10). Given the complexity of the phenotype of T2DM and cognitive dysfunction, we will address the potential pathomechanistic links between the two in the following key areas.

### 2.1 Insulin resistance

All brain cell types express insulin receptors, with the highest densities localized to the olfactory bulb, hypothalamus, hippocampus, cerebral cortex, striatum, and cerebellum (11, 12). Emerging evidence indicates that insulin influences cerebral bioenergetics, enhances synaptic viability and dendritic spine formation, increases the turnover of neurotransmitters, and facilitates clearance of amyloid β peptide while modulating tau phosphorylation (13, 14). T2DM, the predominant form of diabetes mellitus, is generally characterized by chronic hyperglycemia, hyperinsulinemia, dyslipidemia, as well as lipotoxicity, which result in progressive deterioration of insulin secretion and insulin action (15–18). Insulin resistance (IR) is defined as the lack or decreased response of the target tissues to insulin (19, 20). Notably, evidence has shown that peripheral IR results in loss of brain function, which in turn is strongly associated with brain degeneration, cognitive dysfunction, depression, and AD (21–24). Similarly, brain insulin resistance (bIR) can be defined as the failure of brain cells to respond to insulin as they normally would, resulting in impairments in synaptic, metabolic, and immune response functions (25). Individuals with relatively diminished brain insulin sensitivity have a particularly high risk for an AD-like brain pattern (26). Indeed, preclinical and clinical findings support the hypothesis that bIR underlies the basic neuropathological mechanism of cognitive impairment in the aging-related, T2DM-associated, and neurodegenerative context (26). Through these multiple pathways, we hypothesize that insulin resistance could contribute to neurodegeneration, which in turn mediates and promotes the development of AD, vascular cognitive impairment, and other dementias. Notably, metabolites of the intestinal flora, such as bile acids (BAs), short-chain fatty acids (SCFAs) and amino acids (AAs) may influence to some extent the decreased insulin sensitivity associated with T2DM dysfunction and regulate metabolic as well as immune homeostasis (27).

### 2.2 Gut-brain axis dysregulation

The gut microbiome is known for playing a major role in human health as well as being increasingly recognized as being involved in the pathogenesis of metabolic diseases. Accumulating preclinical and clinical data over the past years has shown that alterations in the gut microbiota affect many organs involved in T2DM and the clinical onset of hyperglycemia (28, 29). Multi-omics (OMICs) studies have shown that single-dose streptozotocin (STZ) -induced hyperglycemia (HG) is sufficient to induce and exacerbate intestinal dysbiosis through modulation of the cecum metabolite pool by analyzing the taxonomic composition, transcriptional activity, and small molecule libraries of the cecum (30). A two-stage case-control metagenome-wide association study (MGWAS) based on deep next-generation shotgun sequencing showed that patients with T2DM were characterized by gut microbial dysbiosis, a decrease in the abundance of some universal butyrate-producing bacteria and an increase in various opportunistic pathogens, as well as an enrichment of other microbial functions conferring sulfate reduction and oxidative stress resistance (31). Intestinal dysbiosis promotes insulin resistance and inflammation, exacerbating diabetes; diabetes further worsens the intestinal dysbiosis, creating a mutually reinforcing mechanism. GM dysbiosis synergistically results in (i) a general increase in pro-inflammatory bacteria and a decrease in anti-inflammatory bacteria; (ii) impair intestinal tight junction integrity by increasing production of inflammatory metabolites and intestinal inflammation; and (iii) induced neuroinflammation, accelerated Aβ fibrillogenesis and parenchymal plaque burden (32). Several studies report that a variety of intestinal bacteria responsible for the production of lipopolysaccharides (LPS), a neurotoxin that disrupts paracellular barriers by cleaving intercellular proteins, such as E-cadherin in epithelial cells, leading to the “leaky gut” phenomenon (33, 34). LPS activates microglia and astrocytes, triggering neuroinflammation that promotes amyloid precursor protein accumulation, Aβ42 fibrillogenesis, plaque formation, and ultimately neuronal loss—a key pathway in neurodegeneration (35–37). Dysbiosis of gut microbiota promotes the harmful intestinal substances enter the systemic circulation through a compromised intestinal barrier, triggering systemic inflammation, which in turn destroys the blood–brain barrier and activates the TLR4/NF-κB signaling pathway in the brain, causing neuroinflammation (38, 39).

### 2.3 Oxidative stress and neuroinflammation

An increasing number of studies have indicated that increased oxidative stress is associated with neuronal damage and is a key factor contributing to the onset and progression of DCAD (40–42). ROS are normally produced as by-products of oxygen metabolism, but various factors can elevate its production. Among them, diabetes is a major cause of increased ROS generation by auto-oxidation of glucose, protein glycation, and through the polyol pathway (43). Oxidative stress has been shown to be a major causal factor compromising neuronal loss and synaptic disruption by impairing brain mitochondrial homeostasis, as seen in diabetic mice models, ultimately having deleterious effects on cognitive performance (44). In addition, a wide range of clinical studies have noted that oxidative stress is contribute significantly to the pathogenesis and progression of cognitive dysfunction (23, 45–47). On the other hand, oxidative stress has also been shown to be a facilitator of neuroinflammation, which is another primary contributor to the progression of cognitive decline in T2DM (48).

During T2DM, influenced by HG, microglial activation can exacerbate cytotoxicity and neuronal damage. Previous studies have revealed that in T2DM rat models, microglia activation in the brain is evident, resulting in overexpression of proinflammatory cytokines in the brains of these model rats, along with a marked decline in their learning and memory abilities (49). In contrast, treatment of T2DM model mice with drugs significantly suppressed the over-activation of microglia in the CNS, accompanied by a notable downregulation of pro-inflammatory cytokines and a significant amelioration of cognitive impairment symptom in the mice (50, 51). In the CNS, the functions of microglia are highly dynamic and can adopt different phenotypes based on the microenvironmental signals they receive. This ability to change phenotype, known as polarization, is a key characteristic of microglia, enabling them to adapt to various physiological and pathological conditions (52). However, they are generally classified into two main phenotypes: M1 and M2 (53). M1 phenotype microglia are typically considered pro-inflammatory, playing a key role in immune responses and inflammatory reactions. On the other hand, M2 phenotype microglia are mainly involved in neuroprotection and anti-inflammatory responses. In db/db diabetic mouse model, microglia are polarized into a pro-inflammatory M1 phenotype, along with low levels of a neuroprotective M2 phenotype, and significant cognitive impairment was observed through the Morris water maze test (54). The link between regulatory T (Treg) function and microglia polarization is well established in the brain (55), dipeptidyl peptidase-4 (DPP4)-mediated impairment of Tregs function polarize microglia toward a pro-inflammatory phenotype and subsequently lead to neuroinflammation and cognitive dysfunction in T2DM patients (54). In the T2DM mouse model, the pharmacological intervention inhibited the overactivated microglia and reversed the polarization of microglial phenotypes under T2DM conditions, shifting them from the proinflammatory M1 type to the anti-inflammatory M2 type, with concomitant improvement of cognitive impairment in T2DM mice (56, 57). IF, gut flora dysbiosis, neuroinflammation, and oxidative stress form a self-reinforcing network that underlies DACD.

## 3 Mechanisms of intermittent fasting in neuroprotection

IF is defined as a dietary pattern that restricts the time of eating, rather than the amount or composition of food, in the absence of malnutrition. Popular intermittent fasting diets involve daily time-restricted feeding or intermittent full-day fasting for 2 to 4 days per week. After an 8- to 12-h period of fasting, the liver starts to break down fatty acids to produce ketone bodies, which play a neuroprotective role by improving brain neuronal function, decreasing inflammatory expression and reactive oxygen species (ROS) production, activating brain-derived neurotrophic factor (BDNF) expression in neurons, and restore neuronal metabolism (58, 59). A clinical study in overweight adults suggests that IF increases BDNF levels and may have anti-aging effects (60). Moreover, studies suggest that IF-induced alterations in brain energy metabolism favor the modulation of microglial polarization from the M1 to the M2 phenotype and play an important role in degenerative diseases (61). Studies in diabetic mice have demonstrated that a 28-day IF treatment alleviated diabetes-induced cognitive dysfunction via a microbiota-metabolites-brain axis, benefiting from comprehensive investigations on diabetic mice behavior/synaptic structure, mitochondrial/energy metabolism-related signaling, and an integrated analysis of multi-OMICs (6). Clinical studies on the 5:2 intermittent fasting (IF) and the USDA healthy living (HL) diet have shown that both regimens are effective in improving peripheral IR, lipid metabolism, and cognition (62, 63). Emerging evidence suggests positive relationship between energy limitation, human health and cognition (7). A recent human intervention study showed that IF may influence memory function possibly through modulating adult hippocampal neurogenesis with the potential to be used as an intervention to prevent or boost cognitive decline (64). Ooi and colleagues found that a 3-year IF diet enhanced cognitive functioning in older adults with mild cognitive impairment compared to age-matched adults who irregularly practice IF and age-matched adults who do not practice IF (65). Moreover, in a randomized clinical trial conducted by Kapogiannis et al., both the 5:2 IF regimen and HL diet approaches were effective in reducing brain insulin resistance and improving memory and executive function in in patients with AD, and the improvements were more pronounced in the IF group (66). Interestingly, IF can also reduce the level of circulating insulin in the blood, thereby improving the sensitivity of insulin receptors and upregulating the insulin/IGF-1 signaling pathway, which ultimately enhances the absorption and utilization of glucose by neurons and ameliorates hypometabolism in neurodegenerative disorders (59, 67). In addition, other studies have shown that IF changes the structure of the gut microbiota, increases the abundance of anti-inflammatory bacterial strains, and decreases the level of proinflammatory endotoxins in the gut and serum (68); and leads to increased diversity of gut bacteria, and leads to an increase in the diversity of intestinal bacteria, as well as an enhancement of several antioxidant microbial metabolic pathways (69). Overall, it is strongly hypothesized that IF regimens may be effective in exerting neuroprotection through a variety of pathways, including reduction of insulin resistance, oxidative stress, immune-inflammatory responses, and modulation of intestinal flora dysbiosis, which ultimately ameliorates cerebral energy metabolism and the symptoms of neurocognitive dysfunction.

## 4 Conclusions and future directions

The prevalence of T2DM-associated cognitive dysfunction is increasing due to the extension of the human lifespan, and there is currently no cure. It is important to identify preventive interventions and treatment strategies to ameliorate the progression of neurodegenerative disorders. IF regimens may be effective in exerting neuroprotection through a variety of pathways, including reduction of insulin resistance, oxidative stress, immune-inflammatory responses, and modulation of intestinal flora dysbiosis, which in turn improves symptoms of neurocognitive disorders such as dementia. Pilot study shows neuroprotective effects of IF, but limited research on T2DM-related cognitive dysfunction. In addition, there are no reliable studies indicating the optimal duration of IF programs in T2DM-related research, nor have determined whether there is heterogeneity in IF strategies across individuals and their long-term safety. Considering that diabetic patients are subject to strict glycemic control, the administration of hypoglycemic drugs during IF may cause hypoglycemia and its more serious complications. In addition, specific biomarkers to monitor the efficacy of IF have also not been identified.

Given the existing research gaps in non-pharmacological interventions for DACD, we propose a phased investigation. Systematically translate basic science into generalizable interventions through repeated tests of efficacy and effectiveness. Stage I (Intervention Optimization) will focus on identifying the dose-response relationship of intermittent fasting (IF), including optimal intervention duration (e.g., 12-h vs. 16-h daily fasting) and frequency (e.g., alternate-day vs. 5:2 regimens). Mechanistic outcomes such as insulin sensitivity, inflammatory biomarkers, and feasibility metrics such as adherence rates and adverse events will be prioritized. Stage II (Efficacy Evaluation) will involve a multicenter, randomized, controlled, stratified pilot trial to assess the preliminary efficacy of the optimized IF protocol.
